# Use of family planning and child health services in the private sector: an equity analysis of 12 DHS surveys

**DOI:** 10.1186/s12939-018-0763-7

**Published:** 2018-04-24

**Authors:** Nirali M. Chakraborty, Andrea Sprockett

**Affiliations:** Metrics for Management, 1330 Broadway, Suite 1135, Oakland, CA 94612 USA

**Keywords:** Family planning, Childhood illness, Private sector, Wealth

## Abstract

**Background:**

A key component of universal health coverage is the ability to access quality healthcare without financial hardship. Poorer individuals are less likely to receive care than wealthier individuals, leading to important differences in health outcomes, and a needed focus on equity. To improve access to healthcare while minimizing financial hardships or inequitable service delivery we need to understand where individuals of different wealth seek care. To ensure progress toward SDG 3, we need to specifically understand where individuals seek reproductive, maternal, and child health services.

**Methods:**

We analyzed Demographic and Health Survey data from Bangladesh, Cambodia, DRC, Dominican Republic, Ghana, Haiti, Kenya, Liberia, Mali, Nigeria, Senegal and Zambia. We conducted weighted descriptive analyses on current users of modern FP and the youngest household child under age 5 to understand and compare country-specific care seeking patterns in use of public or private facilities based on urban/rural residence and wealth quintile.

**Results:**

Modern contraceptive prevalence rate ranged from 8.1% to 52.6% across countries, generally rising with increasing wealth within countries. For relatively wealthy women in all countries except Ghana, Liberia, Mali, Senegal and Zambia, the private sector was the dominant source. Source of FP and type of method sought across facilities types differed widely across countries. Across all countries women were more likely to use the public sector for permanent and long-acting reversible contraceptive methods. Wealthier women demonstrated greater use of the private sector for FP services than poorer women.

Overall prevalence rates for diarrhea and fever/ARI were similar, and generally not associated with wealth. The majority of sick children in Haiti did not seek treatment for either diarrhea or fever/ARI, while over 40% of children with cough or fever did not seek treatment in DRC, Haiti, Mali, and Senegal. Of all children who sought care for diarrhea, more than half visited the public sector and just over 30% visited the private sector; differences are more pronounced in the lower wealth quintiles.

**Conclusions:**

Use of the private sector varies widely by reason for visit, country and wealth status. Given these differences, country-specific examination of the role of the private sector furthers our understanding of its utility in expanding access to services across wealth quintiles and providing equitable care.

**Electronic supplementary material:**

The online version of this article (10.1186/s12939-018-0763-7) contains supplementary material, which is available to authorized users.

## Background

Disparities in access to quality healthcare, as well as achievement of health outcomes by wealth status and area of residence has been well documented [1–3]. Poorer individuals are more likely to go without care than wealthier individuals, and to spend proportionately more of their incomes on healthcare [4, 5]. To reduce inequities in health, many public and private health programs in developing countries explicitly aim to serve the poor, often defined as the poorest 40% of the total population or as those living below a given global poverty line. Equity in the utilization of healthcare services, as well as the differences in health outcomes based on socioeconomic status, is of interest to donor organizations, program leaders, researchers, policymakers and governments. Specifically, to ensure progress towards targets 1, 2, 7 and 8 of Sustainable Development Goal 3 of healthy lives and well-being for all, attention must be paid to reproductive, maternal and child health services [6]. Furthermore, the ability to access quality healthcare without financial hardship is a key element of universal health coverage (UHC) [7].

The Every Woman Every Child initiative, the World Health Organization’s (WHO) Partnership for Maternal, Neonatal and Child Health, and the objectives of the Global Financing Facility all focus on family planning (FP) and services for routine childhood illnesses [8]. Diarrheal disease, respiratory infections and malaria currently account for 4,756,000 deaths worldwide across all age groups, while maternal causes account for 230,000 deaths [9]. For children under 5, lower respiratory infections account for 13.06% of deaths, with malaria and diarrhea comprising 10.34% and 8.92% of deaths respectively [9]. Many of these deaths can be averted through the expansion of known interventions and services, such as FP [10], which save the lives of both mothers and children.

Family planning and treatment for diarrhea, malaria and respiratory infection are available at a wide range of health service providers in both the public and private health care sectors. Private providers deliver a significant portion of healthcare services in low- and middle-income countries (LMICs) in both rural and urban areas, for low socioeconomic groups and for the wealthy [4]. But research on the equity of private provision of health care is inconclusive [11]. Some analyses have shown that provision of care in the private sector is inherently inequitable, with significantly greater services accessed by the wealthy [5]. Others have shown that strategies such as contracting out services to the private sector can improve equity, or minimally, that a growing private sector does not harm service delivery equity [12, 13].

Use of the private sector varies by region and service type, highlighting the need to better understand where individuals from different wealth quintiles seek healthcare [14]. In a meta-analysis of Demographic and Health Surveys (DHS) data from 57 LMICs, Campbell and colleagues (2015) find that between 15% and 25% of FP users in the poorest quintile use the private sector across all geographic regions (Sub-Saharan Africa, Middle East/Europe, Asia, Latin America), and between 45% and 50% of those in the wealthiest quintile use the private sector [15]. Yet treatment for childhood illnesses differs from treatment location for FP. Globally, over half of all care for children seeking treatment for diarrhea, fever, and cough is provided by private providers, with more urban and wealthy people using the private sector than rural or poorer individuals [14].

While policymakers have often invested more in public sector service delivery, there is growing interest in how the private sector can complement the public sector [16]. For policymakers and program leaders who are designing and implementing interventions to address causes of maternal, neonatal and childhood mortality and morbidity, it is important to understand where individuals of different wealth and geographic residence seek care. For those with initiatives in multiple countries, it is also important to understand if countries behave similarly with regard to service provider choices. This paper explores use and source of family planning, and source of care for children with diarrhea, fever or cough by residence and wealth quintile to draw meaningful conclusions for private sector healthcare provision.

## Methods

Source of data: This study is a secondary analysis of DHS data regarding source of care for FP services, and childhood diarrhea, fever or cough treatment. The DHS are nationally representative surveys conducted by national implementing agencies with technical assistance from MEASURE DHS and ICF Macro. Countries adapt individual surveys from a model questionnaire, and DHS data files are prepared in a structured way allowing for cross-country comparison [17]. DHS datasets were downloaded from www.measuredhs.com and analyzed using Stata 13/SE. Datasets are publicly available, so no ethical approval was sought.

A purposive sample of twelve countries was selected for a detailed analysis of FP and child health-related health service use. All twelve countries had a DHS survey conducted between 2012 and 2014, and used the DHS phase VI or VII questionnaires. To select countries, the 33 surveys conducted in this timeframe were first compared against the 24 USAID FP priority countries, resulting in 13 overlapping countries [18]. The list of surveys was also compared against the USAID Ending Preventable Child and Maternal Death (EPCMD), and AIDS-free generation countries [19]. Preference was given to countries that appeared on more than one list, but the final set of countries was selected to also have economic and geographic diversity. Table 1 lists the countries included, year of DHS survey, USAID priority list, income status and region.

**Table 1:** Survey year, USAID priority status, Income level, region and sample sizes of data used

Country	Survey Year	USAID priority			Income^a^	Region^b^	Sample size	
		FP	EPCMD	AIDS-free			Women	Children
Bangladesh	2014	✓	✓		L-M	SA	17,863	6679
Cambodia	2014			✓	L-M	EAP	17,578	5799
Democratic Republic of Congo	2013–2014	✓	✓	✓	Low	SSA	18,827	10,701
Dominican Republic	2013			✓	U-M	LAC	9372	2895
Ghana	2014	✓	✓	✓	L-M	SSA	9396	4170
Haiti	2012	✓	✓	✓	Low	LAC	14,287	5176
Kenya	2014	✓	✓	✓	L-M	SSA	31,079	14,537
Liberia	2013	✓	✓		Low	SSA	9239	5072
Mali	2012–2013	✓	✓		Low	SSA	10,424	6439
Nigeria	2013	✓	✓	✓	L-M	SSA	38,948	18,997
Senegal	2014	✓	✓		Low	SSA	8488	4348
Zambia	2013–2014	✓	✓	✓	L-M	SSA	16,411	9038

^a^Income and region data from World Bank [39]. *Low* lower income, *L-M* lower-middle income, *U-M* upper-middle income

^b^*SA* South Asia, *EAP* East Asia and Pacific, *SSA* Sub-Saharan Africa, *LAC* Latin America and Caribbean

Analysis methods: The women’s response file, including data from women of reproductive age (15–49 years), from the full DHS dataset was downloaded for the most recent survey from each country. This file contains key household level variables, including urban or rural residence and wealth quintile. The wealth quintile variable is constructed by using principal components analysis on a list of household possessions and housing characteristics, ranking all individuals by their relative wealth, and then dividing the ranking into equal fifths [20]. Quintile 1 refers to the poorest 20% of people in the country, and quintile 5 refers to the wealthiest 20% of people. The wealth quintile reports a measures of relative wealth, and as such, quintile 1 in one country does not indicate the same level of poverty as quintile 1 in another country. As a relative measure of wealth across countries, DHS data have been previously used in cross-country analyses of equity [14, 21–23].

For FP outcomes, analyses were restricted to current users of modern contraception, based upon the variable for current contraceptive method. The recoded source of most recent resupply was used in analyses [17]. These categories are government clinic/pharmacy, government home/community delivery, NGO, private clinic/delivery, pharmacy, shop/church/friend, other. We combined other with the shop/church/friend category for our analyses.

For outcomes of diarrhea, fever and cough, the DHS asks female respondents about illness in the prior two weeks for each of their children under the age of 5. Our analyses focused on the youngest child under age 5 (the first child listed). For diarrhea, respondents are asked if the child had diarrhea in the previous two weeks, “Did you seek advice or treatment for the diarrhea from any source?”, and “Where did you seek advice or treatment?”. Respondents are also asked if the child had a fever in the previous two weeks, if the child had an illness with a cough in the previous two weeks. If the child had a cough, the respondent is further probed as to whether the child “breathe[s] faster than usual with short rapid breaths or [has] difficulty breathing?”. Children whose cough is accompanied by rapid breathing, and those with fever, are asked about sources of care, if any. In the DHS questionnaire, questions about treatment provided for fever and cough are grouped together. Consequently, in our analyses, we have grouped these two outcomes together, rather than dropping observations for children who experienced both symptoms in the two weeks prior to the survey, or reporting on those responses separately. The symptom of cough with rapid breathing is indicative of an acute lower respiratory tract infection (ARI), and the term ARI is used to describe the data.

For each country, sources of care for diarrhea and fever/acute respiratory infection are entered in up to 24 categories. The categorization within a survey is the same for source of care questions for diarrhea and for fever/ARI. To facilitate analysis, these categories were recoded into the same eight groups for all 12 countries, and are presented as such. Children may seek care at more than one place for the same episode, so analyses presented allow for multiple responses. The groups are: government hospital; government health center; government community health workers, mobile clinics or other public sector sources; private sector hospital, clinic or doctor; private sector pharmacy; NGO or mission (faith based) facility; other private qualified provider; unqualified provider. Examples of country-specific sources of care re-categorized into “other private qualified provider” are “home of trained health worker”, “nurse/auxiliary”, and “private – fieldworker”. Examples re-categorized into “unqualified provider” are “market” “drug peddler”, “street vendor”, “friends/relatives”, and “traditional practitioner”.

Weighted univariate, bi-variate and stratified bi-variate tabulations were conducted for FP, for diarrhea and for fever/ARI. Covariates of interest were wealth quintile and urban/rural residence. To explore whether sources of FP and treatment for childhood illness overlapped, we limited our sample across all 12 countries to mother/child pairs who reported both a source of FP and a source of treatment for her child. We then collapsed the two public facility categories, and the “other” categories from the source of treatment for childhood illness, to make them comparable to the FP sources.

Analyses focused on patterns to identify if there were any trends in use of private sector services, which services or providers within the private sector were more favored, and if it was possible to assess these data by wealth quintile. Table 1 lists the sample sizes for each of the 12 countries for both FP and childhood diarrhea and fever/ARI services. Sample sizes varied greatly among countries, indicative of varying illness prevalence, as well as different survey requirements. For example, in Nigeria and Kenya, large samples were collected in order to have region and district-specific estimates for key indicators. Analyses by multiple sub-categories were limited by small sample sizes in some instances.

## Results

### Family planning

The modern contraceptive prevalence rate (mCPR) varied widely across the 12 comparison countries, from 8.1% in the Democratic Republic of Congo (DRC) to 52.6% in the Dominican Republic (Table 2). The mCPR generally rose with increasing wealth, with the interesting exceptions of Cambodia and the Dominic Republican where the reverse trend was seen with higher use of modern contraceptive methods in the lowest two wealth quintiles (Table 2). In Kenya, the mCPR was about 40% in all quintiles except the lowest where only 23.5% of all women were using modern methods. And in Bangladesh we see that approximately half of all women, regardless of wealth quintile, used a modern method of contraception.

**Table 2:** Modern contraceptive prevalence by wealth quintile (%), by location (%), and overall

Country	Wealth Quintile					Urban	Rural	mCPR (%)
	Q1	Q2	Q3	Q4	Q5			
Bangladesh	50.8	52.2	52.0	50.0	50.2	52.4	50.4	51.0
Cambodia	29.2	31.0	26.9	26.3	21.1	19.3	28.3	26.6
DRC	3.6	5.0	5.0	11.2	14.0	13.1	5.0	8.1
Dom. Rep.	55.7	60.0	52.1	48.6	47.9	51.9	54.8	52.6
Ghana	17.8	20.5	21.0	16.5	15.8	16.0	20.6	18.2
Haiti	20.8	20.3	24.4	23.3	19.3	21.4	21.8	21.6
Kenya	23.5	40.0	43.0	44.4	40.7	41.5	37.5	39.1
Liberia	13.5	16.5	21.8	25.4	22.9	22.8	16.9	20.5
Mali	3.2	4.8	5.8	12.3	19.6	18.6	6.7	9.6
Nigeria	0.9	4.3	10.1	15.3	22.2	16.7	7.1	11.1
Senegal	9.7	11.2	14.5	19.2	17.0	18.3	10.7	14.7
Zambia	24.8	31.6	35.8	35.1	33.6	34.7	30.5	32.5

**Fig. 1:**
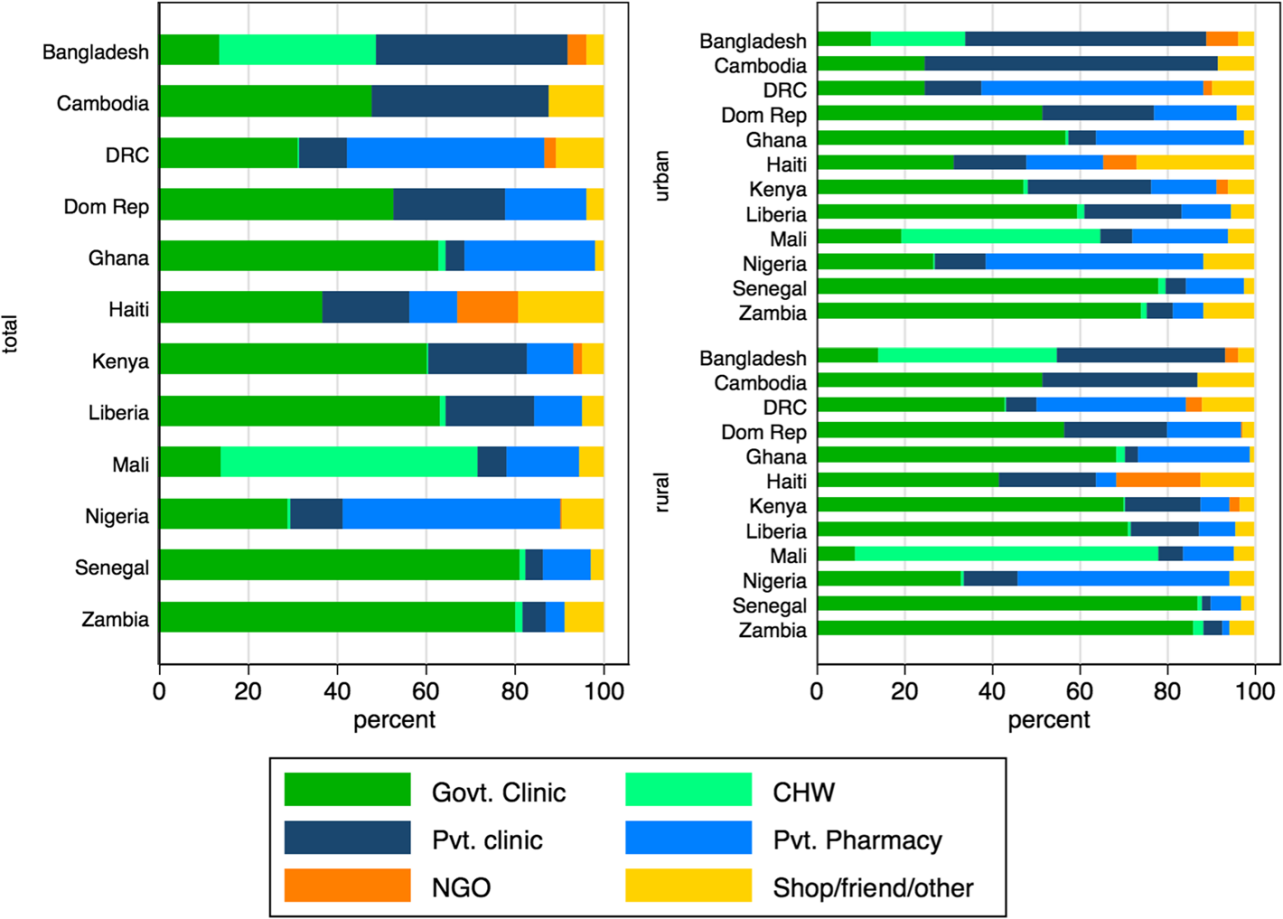
Source of family planning by country and location. *Data source:* DHS surveys conducted between 2012 and 2014 in 12 priority countries listed in Table 1

When exploring the type of public and private sector sources used for FP services, patterns across countries differed (Fig. 1). In Bangladesh and Cambodia, private clinics provided about 40% of FP services, and nearly 50% of services in Nigeria were reported as provided through private pharmacies. Bangladesh and Mali both had a large proportion of services provided by community health workers in the public sector, at 35.3% and 57.8%, respectively. In Kenya, 60.2% of services were provided by government clinics, with 34.6% provided by private clinics, pharmacies, or NGOs.

NGO services accounted for a limited amount of FP service delivery across the six types of care sources providing less than 4% of care in most countries, except for nearly 20% of rural care in Haiti. Haiti was also notable for its comparatively high use of shop/friend/other for FP services (26.8% of FP services in urban locations and 12.4% in rural). In other countries, patterns in the types of facilities used for FP services remained similar across urban and rural divides, although private clinics were more often used in urban settings than in rural.

**Fig. 2:**
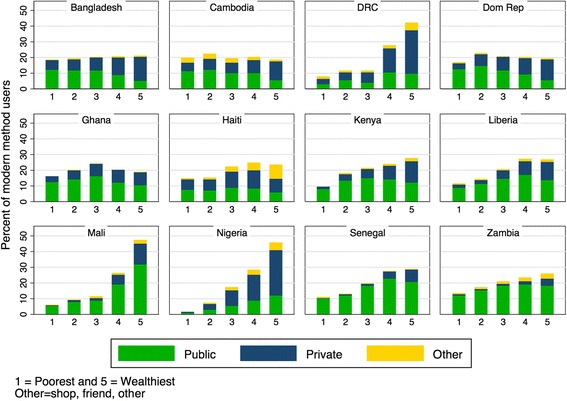
Source of modern contraceptives by wealth quintile. *Data source:* DHS surveys conducted between 2012 and 2014 in 12 priority countries listed in Table 1

Figure 2 shows the distribution of all modern method users across wealth quintiles, and where they went the last time they needed FP. As seen in Fig. 2, the private sector was a more significant source of FP for women in quintiles 4 and 5, as compared to poorer wealth quintiles. In general, use of the private sector increased by wealth quintile. Even in countries where a similar percentage of women in each wealth quintile used FP, wealthier women sought their services from the private sector.

### Diarrhea

Worldwide, treatment patterns for diarrhea varied, despite few differences in the prevalence rates. Focusing on the 12 comparison countries, prevalence of diarrhea in the previous two weeks ranged from 5.9% in Bangladesh to 24.8% in Liberia and Senegal (Table 3). The prevalence of diarrhea in Bangladesh was low (4.6–6.7%) across all wealth quintiles. The Dominican Republic had the largest difference in prevalence across quintiles where 23.2% of the lowest wealth quintile reported diarrhea versus 12.9% in the highest wealth quintile. Overall, diarrhea prevalence was comparable between urban and rural locations; in Liberia, this difference was largest where 22.1% was reported in urban settings and 27.8% in rural settings.

**Table 3:** Diarrhea prevalence by wealth quintile, location and overall

Country	Wealth Quintile					Urban	Rural	Prevalence(%)
	Q1	Q2	Q3	Q4	Q5			
Bangladesh	6.5	6.7	6.1	4.6	5.6	5.9	5.9	5.9
Cambodia	17.4	12.3	10.9	14.5	12.3	13.7	13.5	13.6
DRC	21.0	20.2	17.9	23.7	21.7	22.0	20.3	20.9
Dom. Rep.	23.2	23.6	18.8	14.5	12.9	19.4	18.1	19.1
Ghana	16.1	16.3	13.7	10.5	6.5	10.9	14.2	12.7
Haiti	21.0	26.8	26.2	24.4	16.7	23.5	23.2	23.3
Kenya	21.3	19.8	17.4	16.1	11.7	15.5	18.1	17.1
Liberia	30.2	26.2	21.9	23.8	20.3	22.1	27.8	24.8
Mali	8.8	9.4	11.8	10.7	11.1	10.5	10.3	10.3
Nigeria	14.8	14.3	11.9	10.7	8.8	11.2	12.9	12.3
Senegal	29.0	25.2	20.8	24.2	24.5	23.7	25.8	24.8
Zambia	18.2	18.6	18.9	22.3	16.9	20.1	18.3	19.0

**Fig. 3:**
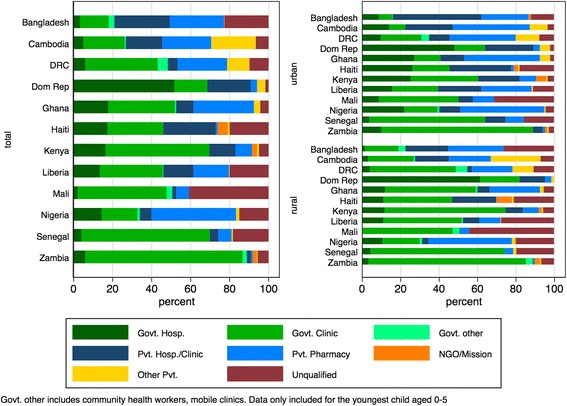
Source of diarrhea treatment by country and location. *Data source:* DHS surveys conducted between 2012 and 2014 in 12 priority countries listed in Table 1 *Notes:* Only includes children for whom care was sought

Among children for whom treatment was sought, public health centers provided the most care in many countries, ranging from 34% of all care in Liberia to 83% in Zambia (Fig. 3). Children in Mali visited an unqualified provider in 42% of cases, but in the Dominican Republic unqualified providers were visited in only 1.5% of cases. The private sector and NGO sectors provide the majority of treatment to urban patients in seven of the 12 countries, however in rural areas, only Bangladesh, Cambodia and Nigeria have such a preponderance of private sector utilization. Overall, government hospitals and clinics were widely used, although clinics were more often visited than hospitals. The Dominican Republic presents the exception, where 48.1% of children who sought care visited a government hospital and another 15.9% visited a government clinic. A substantial proportion of children did not receive any treatment for their most recent episode of diarrhea, ranging from 22.2% in Cambodia to 55.9% in Senegal (Additional file 1: Table S1).

**Fig. 4:**
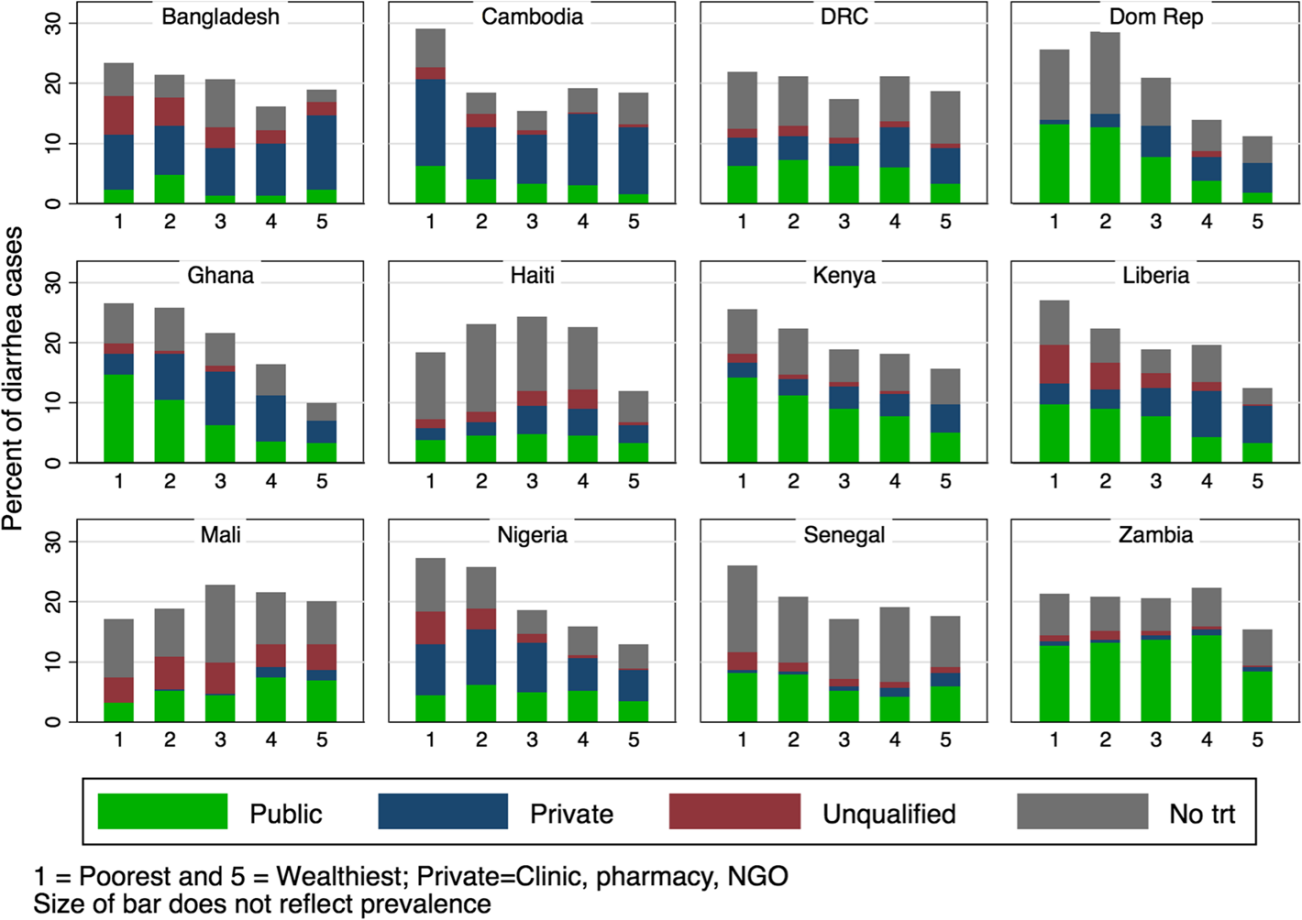
Source of diarrhea treatment by wealth quintile. *Data source:* DHS surveys conducted between 2012 and 2014 in 12 priority countries listed in Table 1

Across all 12 countries, over half of all cases of diarrhea occurred in the lowest two quintiles versus just over one-quarter of cases in the highest two wealth quintiles (Fig. 4). Although one-third of children in Kenya did not seek treatment for diarrhea, all wealth quintiles were more likely to use public services than private. This difference was more pronounced in the lowest wealth quintile. In Cambodia, where nearly 80% of children across all wealth quintiles seek treatment for diarrhea, the trend for use of public or private services is reversed. All wealth quintiles were more likely to use private rather than public services; 61.2% of diarrhea cases amongst the highest wealth quintile visited private services compared to 50.1% in the lowest quintile. In Senegal, only 1.9% of cases in the lowest wealth quintiles visited a private provider compared to 12.4% in the highest wealth quintile.

### Fever/ARI

Across all 12 countries, between 12 to 48% of children were reported to have had fever or cough, classified as ARI, in the last two weeks (Table 4). The prevalence of fever/ARI was generally higher in the lowest two wealth quintiles as compared to the highest two wealth quintiles. In the Dominican Republic, reported cases of fever/ARI by quintile were 36% in Q1 compared to 22.4% in Q5, followed by Bangladesh with 41.8% prevalence in the lowest wealth quintile and 34.7% prevalence in the highest wealth quintile. Mali reported the lowest prevalence of fever/ARI with just over 10% across all wealth groups. Prevalence by urban/rural location was similar to overall prevalence; the largest difference was in Kenya where 30.1% prevalence was reported in urban settings and 37.0% in rural settings.

**Table 4:** Prevalence of cough or fever by wealth quintile, location and overall

Country	Wealth Quintile					Urban	Rural	Prevalence(%)
	Q1	Q2	Q3	Q4	Q5			
Bangladesh	41.8	40.6	44.7	39.8	34.7	37.5	41.3	40.3
Cambodia	33.1	27.6	29.9	30.1	30.6	30.6	30.3	30.3
DRC	38.6	37.8	38.0	44.0	32.9	37.0	38.8	38.3
Dom. Rep.	35.9	31.4	30.2	31.3	22.4	31.6	27.7	30.6
Ghana	19.6	22.1	20.8	15.7	16.7	17.1	20.6	19.0
Haiti	47.0	49.6	51.8	47.4	43.7	46.6	49.0	48.1
Kenya	36.2	41.1	37.0	34.6	24.7	30.1	37.0	34.4
Liberia	39.6	36.1	32.0	34.7	33.4	32.9	38.1	35.3
Mali	12.7	12.3	11.1	11.5	11.2	11.7	11.8	11.8
Nigeria	16.2	17.2	17.3	14.6	12.4	14.9	16.1	15.6
Senegal	17.7	14.8	10.1	17.6	20.2	17.6	14.7	16.0
Zambia	28.5	29.9	26.5	23.8	22.3	23.4	28.3	26.4

**Fig. 5:**
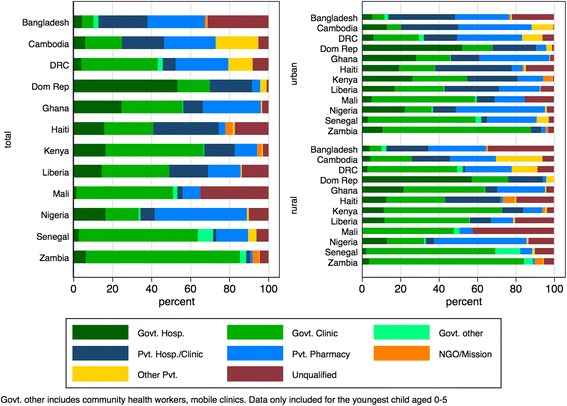
Source of ARI/fever treatment by country and location *Data source:* DHS surveys conducted between 2012 and 2014 in 12 priority countries listed in Table 1 *Notes:* Only includes children for whom care was sought

For fever and ARI, the private sector dominated care in Bangladesh, Cambodia and Nigeria, while providing a very small proportion of care in Senegal, Zambia and Mali. A significant proportion of ill children sought care from an unqualified provider, with the highest proportions in Mali (36%) and Bangladesh (33%). These patterns are similar to care-seeking for diarrhea, and provide insight into use of the private sector for childhood illness worldwide. Among cases, public providers were a more common source of care in Zambia (61.9%) while private providers were visited most often in Cambodia (64.2%) (Fig. 5). Children who did not seek care for fever/ARI ranged from 13.1% in Cambodia to 56.9% of children who did not seek care in Haiti (See Additional file 1: Table S1).

**Fig. 6:**
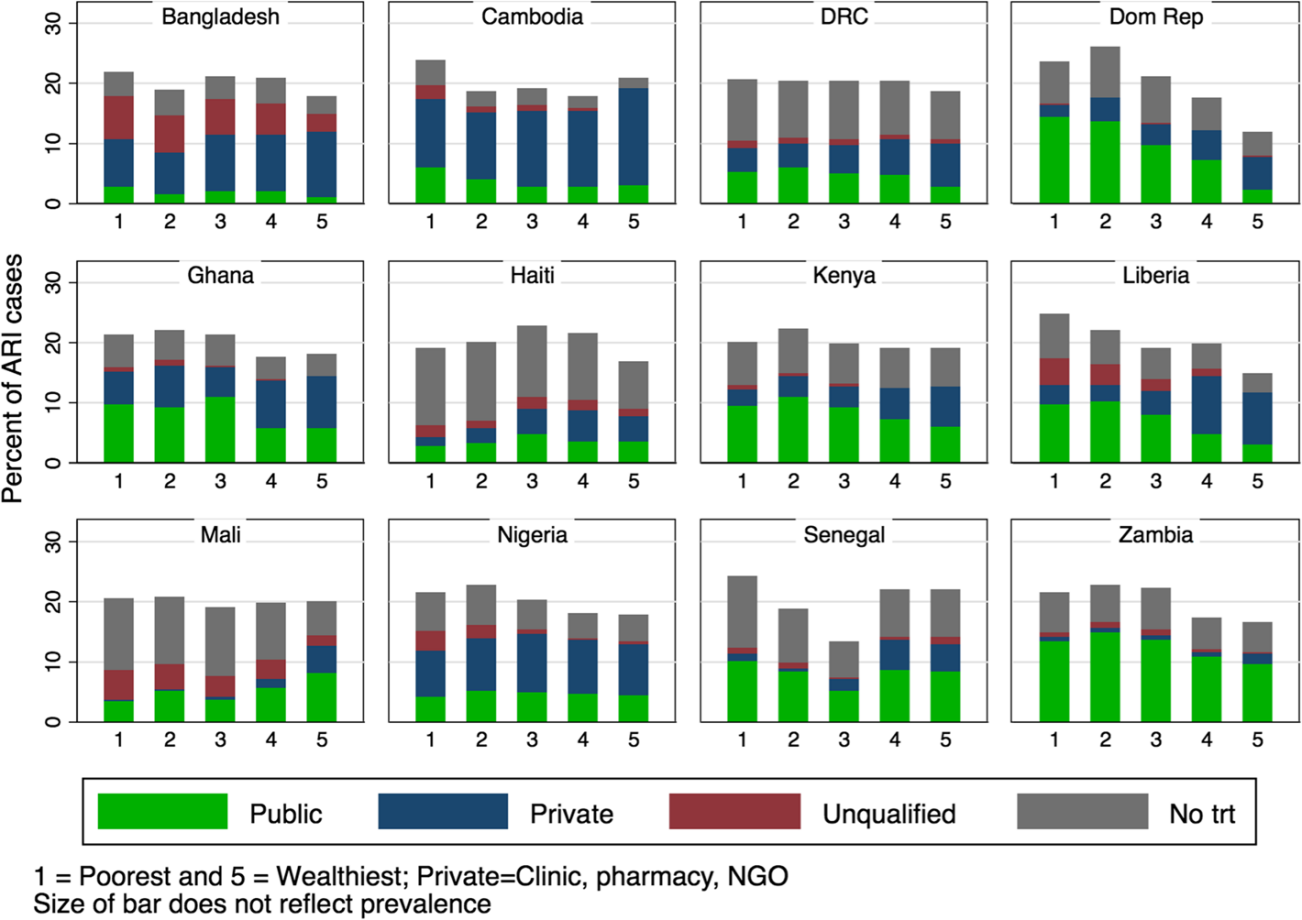
Source of ARI/fever treatment by wealth quintile *Data source:* DHS surveys conducted between 2012 and 2014 in 12 priority countries listed in Table 1

Figure 6 indicates how cases of fever/ARI were distributed across wealth quintiles and source of care. Of all cases of fever/ARI in the Dominican Republic, 25.4% are found in households in the poorest quintile, while 12.0% are in the wealthiest households. In contrast, in the DRC, cases of fever/ARI are fairly evenly distributed across wealth quintiles. In Haiti and Mali, a large proportion of fever/ARI cases in the poorest quintile received no treatment (65.0% and 54.1%, respectively). In Cambodia, where most cases received care, 48.8% of the poorest quintile and 82.6% of the richest quintile used either a private hospital/clinic/doctor, private pharmacy, or other qualified private provider. In Senegal, where 57.4% of cases received care, use of a private provider also similarly rose as wealth quintiles increased (4.9% of the lowest wealth quintile compared to 20.7% of the highest wealth quintile).

### Sources of family planning and childhood illness treatment

Among mother/child pairs who reported both a source of FP and a source of treatment for diarrhea, 75.4% of women who went to a public facility for FP also sought care for their child at a public facility (See Additional file 1: Table S2). In contrast, only 30.5% and 22.4% of women who went to a private facility and private pharmacy, respectively, for FP returned to a similar source type for child diarrhea treatment. Across all sources of FP, women most often sought care for their child with diarrhea from a public facility. A similar pattern was observed when comparing sources of FP and care for childhood fever/ARI (See Additional file 1: Table S3). However, among women who went to a private facility for FP, the most common type of facility women visited for their child with fever/ARI was also a private facility (34.4%).

### Limitations

It is important to note the following limitations when using DHS or other household data. All data is self-report, and in the case of FP services, the recall time may be quite long. Women may mis-specify the source of care for themselves or their children. Second, for childhood illness episodes, research indicates that care may be sought at more than one location, and that the reason for seeking care at multiple locations is important [22]. The DHS does not capture the order of care-seeking, nor the reason for choosing a particular site. With regard to the analyses presented, one limitation in the childhood illness analyses is that only data from the youngest child under 5 years of age was used (only 1 child per household). However, it is possible that there are multiple children under 5 in a household who experience an illness simultaneously, and care sought for one child may be influenced by care sought for another. Additionally, recall of treatment between children may be subject to error. For comparability of analyses across FP and diarrhea and fever/ARI care-seeking, all data were weighted using household weights since wealth is a household level variable, rather than ensuring data weighted to be representative of all women, or all children under 5.

## Discussion

To measure progress towards Sustainable Development Goal 3, the global community has agreed upon a number of targets. Notably, we will dramatically reduce maternal mortality, end preventable deaths of children under five, and ensure universal access to reproductive health services, while attaining universal health coverage [6]. Given the already high reliance on the private sector for provision of health services in many countries, it is important to determine if support or promotion of the private sector will increase inequity in access to health services [24]. This concern exists because private services are not free, whereas public sector services in many countries are free or subsidized.

The source of care for FP varied significantly across countries when looking at our 12 country sample, with the private sector providing the majority of FP in Bangladesh, Cambodia, DRC, Haiti and Nigeria, and was the source of just under 50% of contraceptives in the Dominican Republic. The DRC, Nigeria, Mali and Senegal all exhibit a similar pattern of contraceptive use with low overall mCPR, and contraceptive use highly skewed towards the wealthiest women. However, our analyses provide further insight, demonstrating that even the wealthy women in Senegal, Mali and Zambia have limited reliance upon the private sector. In these three countries in particular, the private sector is not highly utilized for any health need examined in this article, indicating a generally small private sector footprint.

Limited use of the private sector for FP means that interventions that want to leverage these private providers successfully are non-traditional. One example from Mali is the creation of a public sector social franchise network, where selected primary health facilities receive branding, training and oversight from a social franchisor [25]. By contrast, in Nigeria Shelton and Finkle [26] advocate for expansion of traditional social franchising and social marketing channels, as well as task-sharing so that private patent medical vendors can deliver injectable contraceptives.

The introduction of a new contraceptive method is a good example of the utility of looking at FP use by wealth and source of supply across countries. In the DRC, the Dominican Republic, and Nigeria, private sector pharmacies are the largest provider of short-term methods. Although pharmacies are primarily providing oral contraceptives in these countries, it is likely that the role of pharmacies will expand with the introduction of Sayana® Press in DRC and Nigeria, as well as social marketing of Sayana Press in Nigeria, and significant purchases in Senegal. Of the 12 countries in this study, nine have received some doses of this new contraceptive between 2014 and 2017. Among the largest procurers are Senegal, DRC and Nigeria receiving 761,200 doses, 818,000 doses, and 2,508,000 doses respectively^1^ [27]. The private sector will likely play a very large role in providing access to this product, and our data show that women across the socio-economic spectrum use the private sector to obtain their FP method.

When examining the treatment of childhood illnesses, it is important first to understand patterns of prevalence. Our data show that in the Dominican Republic, Ghana, Haiti, Kenya, Liberia and Nigeria, household wealth quintile and diarrhea prevalence have a high inverse correlation. However, only Ghana, Kenya and Liberia have a greater than two percentage point difference in prevalence between urban and rural areas. Combining these two findings, it is indicative of the fact that the poorest are primarily located in rural areas in these countries. Prevalence of fever/ARI is only consistently associated with household wealth in Kenya and Zambia, and in both of these countries, prevalence of fever/ARI is lower in the poorest quintile than in quintile 2. There is a greater than two percentage point difference in prevalence between urban and rural areas in Bangladesh, Ghana, Haiti, Kenya, Liberia, Zambia, Dominican Republic and Senegal, with the latter two countries having a higher prevalence in urban areas.

In our 12 country sample, the Asian countries and Nigeria had substantially greater use of the private sector for treatment of both diarrhea and fever/ARI, consistent with trends seen across all LMICs [15]. Among those who seek treatment for diarrhea or fever/ARI, use of the private sector is positively correlated with household wealth quintile, even in countries with an overall low reliance upon the private sector. Patterns differ by type of private sector facility, with less than 10% of sick children in the poorest quintile visiting a private clinic in Kenya, Haiti and Liberia, compared with 25%–40% of those in the richest quintile in those same countries. Use of private sector pharmacies, by contrast, is much more equitable, especially in the Dominican Republic, Ghana, Kenya and Nigeria. However, many childhood illnesses are not treated in the formal medical sector, revealing an unmet need for quality care.

Across the 12 comparison countries, more than one-third of all children did not receive treatment for diarrhea. Over 50% of children with fever/ARI in Haiti and Mali received no treatment, or treatment from an unqualified source. The proportion of children who received no treatment for fever/ARI was strongly negatively correlated with wealth status in Haiti, Liberia, Mali and Senegal, with a similar finding for no treatment for diarrhea only present in Haiti. It can be argued that not all episodes of diarrhea or fever require formal medical care. In fact, promotion of appropriate home treatment of diarrhea with oral rehydration solution has been part of global recommendations since the 1970s, and has been estimated to reduce 93% of all diarrheal mortality in children under 5 with 100% coverage [28]. However, given that almost 40% of under 5 mortality is caused by diarrheal disease, respiratory infection and malaria, the inequity in the proportion of children who do not receive any treatment necessitates further investigation [29].

The private sector is not a homogeneous entity. It contains different types of providers, ranging from formal medical clinics, to registered pharmacies, drug sellers, general retailers, faith-based and non-governmental organizations, and can be for profit or non-profit. As we have seen in our analyses, there is substantial variation in outpatient care-seeking through the private sector across countries [15, 22, 30]. A deeper examination of the total relevance of the private sector in providing care for specific services, plus the relative usage of the private sector by wealth group and urban/rural residence, can help policymakers direct resources towards improving coverage for needed interventions.

Several systematic reviews have examined the use of the private sector in outpatient health services, including whether private sector providers improve coverage, have quality services or provide services efficiently and equitably [31–34]. Others have examined inequity in health services provision, with notable efforts such as the Countdown to 2015 or meta-analyses of national household survey data for inequity, specifically for child health or FP services [1, 5, 35, 36]. Few have combined both lines of inquiry, and investigated the use of the private sector by wealth status [13, 14, 21]. Of these, Campbell et al., and Grepin aggregate data from 57 and 70 countries, respectively, masking variation, while Hotchkiss focuses only on contraceptives. Our analyses focused on two types of services which are routinely provided in the private sector, and which are services that usually require a payment. Data is disaggregated by country to allow for more nuanced analyses. Improvement in use of FP and diarrhea and fever/ARI services for those in need, who have been shown to be more likely to be poor, will lead to lower mortality and morbidity among women and children [1, 28, 37, 38].

## Conclusion

This investigation of type of care sought by wealth, residence and reason for need across 12 countries combines information often found in siloes, such as only looking at FP care seeking, or investigating wealth inequities without addressing type of care sought. Overall, parents were more likely to take their children to public sector services for the treatment of childhood illnesses across all wealth quintiles. However, parents chose the private sector more often for treatment of fever/ARI than for diarrhea. Reasons would have to be explored, but present an opportunity to increase use of the same private sector facilities for diarrhea treatment, as well as among those seeking no treatment across all wealth quintiles. Contrasted to diarrhea and fever/ARI services, use of public and private sector services for FP varied substantially country by country, and use of modern contraception was not correlated with the use of public or private services. Furthermore, there was little correlation between use of the private sector for childhood illness, and use of the private sector for FP in the same household (between a woman and her child). This secondary analysis of DHS data demonstrated differences in where individuals from different wealth quintiles sought FP services and care for children with diarrhea and fever/ARI. Additional research to understand why motivations for seeking private services differ for FP and childhood illness may help to inform strategies to improve coverage of care.

In an era where policymakers, ministry officials and program leaders are continually pressed to do more with less, while simultaneously striving to implement UHC and supporting policies that improve access without causing financial hardship, it is important to understand where individuals of different socioeconomic status seek care for different health services. Being able to leverage a successful program to increase use of family planning through investments in the private sector along with improvements in appropriate care for children can stretch funds for greater results. Similarly, advocating for increased availability of a range of family planning methods at the pharmacies where treatment for diarrhea and fever are sought may improve access for women. Coordination across public and private sectors may help to streamline and maximize the benefits of the services. Examining the role of the private sector furthers our understanding of its utility in expanding access to services across wealth quintiles and providing equitable care.

## Additional file

Additional file 1: **Table S1.** Proportion of ill children who did not receive treatment, by condition and location. **Table S2.** Sources of FP and Diarrhea Treatment (Tx). **Table S3.** Sources of FP and fever/ARI treatment (Tx). (DOCX 21 kb)

## Abbreviations

AIDS: Acquired Immunodeficiency Syndrome
ARI: Acute respiratory illness
CHW: Community health worker
DHS: Demographic and Health Surveys
Dom. Rep.: Dominican Republic
DRC: Democratic Republic of Congo
EAP: East Asia and Pacific
EPCMD: Ending Preventable Child and Maternal Death
FP: Family planning
LAC: Latin America and Caribbean
LARCs: Long-acting reversible contraceptives
L-M: Lower-middle income
LMIC: Low- and middle-income countries
mCPR: Modern contraceptive prevalence rate
NGOs: Nongovernmental organizations
SA: South Asia
SSA: Sub-Saharan African
UHC: Universal health coverage
U-M: Upper-middle income
USAID: United States Agency for International Development
WHO: World Health Organization

## Footnotes

1 UNFPA’s RHInterchange database provides information on number of doses, date of purchase, and recipient. Using this information, author determined introduction of Sayana Press, channel of delivery, and doses.
