# Supplementary material for: Use of family planning and child health services in the private sector: an equity analysis of 12 DHS surveys
Source: Int J Equity Health. 2018 Apr 24;17:50. doi: 10.1186/s12939-018-0763-7 (PMC5916835; doi:10.1186/s12939-018-0763-7)
Supplement: Supplementary file 1 — Table S1. Proportion of ill children who did not receive treatment, by condition and location. Table S2. Sources of FP and Diarrhea Treatment (Tx). Table S3. Sources of FP and fever/ARI treatment (Tx). (DOCX 21 kb) [file 12939_2018_763_MOESM1_ESM.docx]

| Table S1: Proportion of ill children who did not receive treatment, by condition and location | | | | | | |
| --- | --- | --- | --- | --- | --- | --- |
|  | Diarrhea | | | Fever/ARI | | |
| Country | Overall | Urban | Rural | Overall | Urban | Rural |
| Bangladesh | 23.2 | 16.5 | 25.6 | 18.9 | 7.9 | 29.7 |
| Cambodia | 22.2 | 20.9 | 22.5 | 13.1 | 16.6 | 17.9 |
| DRC | 40.3 | 43.2 | 38.9 | 45.7 | 35.9 | 35.7 |
| Dom. Rep. | 43.2 | 44.8 | 38.3 | 32.6 | 37.6 | 27.8 |
| Ghana | 27.8 | 31.9 | 25.1 | 23.0 | 28.6 | 17.3 |
| Haiti | 54.0 | 50.4 | 56.2 | 56.9 | 49.7 | 50.1 |
| Kenya | 32.6 | 33.2 | 32.3 | 34.3 | 23.5 | 27.1 |
| Liberia | 26.0 | 25.6 | 26.4 | 25.7 | 16.8 | 19.8 |
| Mali | 46.3 | 33.9 | 49.7 | 49.9 | 33.1 | 48.9 |
| Nigeria | 28.9 | 28.8 | 28.9 | 26.9 | 15.4 | 22.2 |
| Senegal | 55.9 | 58.5 | 53.8 | 42.6 | 40.3 | 35.4 |
| Zambia | 30.6 | 33.1 | 29.0 | 30.0 | 21.4 | 22.3 |

**Additional File 1**

| Table S2: Sources of FP and Diarrhea Treatment (Tx) | | | | | | | |
| --- | --- | --- | --- | --- | --- | --- | --- |
|  | | | | | |  |  |
|  | Diarrhea Tx Source | | | | | |  |
| FP Source | Public facility | CHW | NGO/ mission | Pvt clinic | Pvt Pharmacy | Other | Total |
| Public facility | 75.4 | 0.8 | 0.9 | 7.2 | 9.2 | 6.5 | 100.0 |
| CHW | 38.3 | 4.5 | 0.0 | 12.3 | 19.4 | 25.6 | 100.0 |
| NGO/mission | 44.3 | 0.0 | 11.2 | 21.0 | 4.9 | 18.6 | 100.0 |
| Pvt clinic | 32.9 | 0.4 | 3.1 | 30.5 | 17.3 | 15.9 | 100.0 |
| Pvt Pharmacy | 52.8 | 1.1 | 0.1 | 13.2 | 22.4 | 10.5 | 100.0 |
| Other | 50.9 | 1.0 | 1.7 | 12.9 | 10.3 | 23.1 | 100.0 |
| Total | 61.2 | 0.8 | 1.5 | 13.4 | 12.5 | 10.5 | 100.0 |

Note: n=3066 (among women who reported an FP source and Tx source for child with diarrhea)

| Table S3: Sources of FP and fever/ARI treatment (Tx) | | | | | | | |
| --- | --- | --- | --- | --- | --- | --- | --- |
|  | Fever/ARI Tx Source | | | | | |  |
| FP Source | Public facility | CHW | NGO/ mission | Pvt clinic | Pvt Pharmacy | Other | Total |
| Public facility | 70.9 | 0.8 | 0.9 | 7.8 | 13.2 | 6.3 | 100.0 |
| CHW | 20.3 | 3.2 | 0.8 | 14.7 | 32.0 | 29.1 | 100.0 |
| NGO/mission | 32.0 | 0.4 | 8.8 | 28.3 | 13.5 | 17.1 | 100.0 |
| Pvt clinic | 23.6 | 0.6 | 2.8 | 34.4 | 21.2 | 17.4 | 100.0 |
| Pvt Pharmacy | 44.2 | 1.4 | 0.3 | 17.8 | 29.8 | 6.6 | 100.0 |
| Other | 42.3 | 2.3 | 0.4 | 17.8 | 15.7 | 21.6 | 100.0 |
| Total | 50.8 | 1.1 | 1.5 | 16.8 | 18.1 | 11.7 | 100.0 |
| Note: n=7952 (among women who reported an FP source and Tx source for child with fever/ARI) | | | | | |  |  |
